# Sustainable Sheep Wool/Soy Protein Biocomposites for Sound Absorption

**DOI:** 10.3390/polym14235231

**Published:** 2022-12-01

**Authors:** Marta Urdanpilleta, Itsaso Leceta, Pedro Guerrero, Koro de la Caba

**Affiliations:** 1 BIOMAT Research Group, University of the Basque Country (UPV/EHU), Department of Applied Physics, Escuela de Ingeniería de Gipuzkoa, Plaza de Europa 1, 20018 Donostia-San Sebastián, Spain; 2 BIOMAT Research Group, University of the Basque Country (UPV/EHU), Department of Applied Mathematics, Escuela de Ingeniería de Gipuzkoa, Plaza de Europa 1, 20018 Donostia-San Sebastián, Spain; 3 BIOMAT Research Group, University of the Basque Country (UPV/EHU), Department of Chemical and Environment Engineering, Escuela de Ingeniería de Gipuzkoa, Plaza de Europa 1, 20018 Donostia-San Sebastián, Spain; 4 BCMaterials, Basque Center for Materials, Applications and Nanostructures, UPV/EHU Science Park, 48940 Leioa, Spain; 5 Proteinmat Materials SL, Avenida de Tolosa 72, 20018 Donostia-San Sebastián, Spain

**Keywords:** biocomposites, sound absorption, sheep wool

## Abstract

The wool fibers of the Latxa sheep breed were combined with a soy protein isolate (SPI) matrix to develop sustainable biocomposites with acoustic properties, adding value to Latxa sheep wool, which is currently considered a residue. Samples with 7, 10, 15, and 20 wt % wool were prepared by freeze drying in order to develop porous structures, as shown by SEM analysis. Additionally, XRD analysis provided the evidence of a change toward a more amorphous structure with the incorporation of wool fibers due to the interactions between the soy protein and keratin present in wool fibers, as shown by the relative intensity changes in the FTIR bands. The biocomposites were analyzed in a Kundt’s tube to obtain their sound absorption coefficient at normal incidence. The results showed an acoustic absorption coefficient that well-surpassed 0.9 for frequencies above 1000 Hz. This performance is comparable to that of the conventional synthetic materials present in the market and, thus, sheep wool/SPI biocomposites are suitable to be used as acoustic absorbers in the building industry, highlighting the potential of replacing not only synthetic fibers but also synthetic polymers, with natural materials to enhance the sustainability of the building sector.

## 1. Introduction

The increasing consciousness about the sustainability agenda has reached a point at which many consumers realize that the culture of using renewable resources with a sustainable life cycle must permeate all spheres of human production. Therefore, they are demanding solutions that are respectful with the environment. Increasing efforts in this regard have led to the development of materials based on natural and renewable materials in fields such as building construction.

Some plant-derived waste, such as petiole, esparto, wood sawdust, and sunflower stem; and animal-derived waste, such as chicken feathers and sheep wool, have been analyzed for potential use as sound insulation panels. The results for these materials have shown good acoustic performance (absorption coefficient values of 0.6–0.9 at medium frequencies) [1,2]. Additionally, natural fibers, such as hemp, kenaf, cotton, and coconut, have shown good sound absorption coefficients, especially at medium and high frequencies [3]. These panels have a fibrillary microstructure, with the presence of empty and wavy cells, which ensures high porosity and high absorption coefficients. In this regard, in sound-absorbing models, absorption coefficients are related to the porosity, tortuosity, and airflow resistance [3].

In this globalized world, products travel very long distances to reach far away markets, with their transport requiring large amounts of fossil fuels, although there are local producers that have a lower carbon footprint, but have to face many economic difficulties to survive. This is the case of the shepherds of Latxa sheep, which preserve landscapes, ecosystems, and biodiversity in the regions of the Basque Country and Navarre (Spain). In the past, this sheep wool was considered a valuable material for the textile industry, even if it had a large diameter that made it coarse [4]. However, with the popularization of synthetic fibers and the changes in consumer demands, these producers do not receive any income for wool after the annual shearing, and they must pay for their wool to be collected and treated; therefore, wool is considered a residue. This problem affects more and more shepherds all over the world. In recent years, efforts have been made to revalorize sheep wool as a fertilizer or compost [5], but we still need to find new applications in the market to convert sheep wool into an alternative material in the context of the circular economy.

Because sheep wool displays very good acoustic and thermal properties, it can be used in the building industry [6]. One fundamental key to understanding the excellent properties of sheep wool as an acoustic insulator is its fibrous structure. Sheep wool is a very poor insulator on its own, but it has very good sound-absorbing properties when combined with rigid sheets (e.g., two brick layers), displaying a good capacity to reduce mechanical coupling between sheets and, thus, decreasing sound transmission through the system.

In addition to its insulating properties, sheep wool has low flammability and a higher inflammation point than cotton or many synthetic fibers. Furthermore, it has the capacity to extinguish flames once the fire source is extinguished [7]. The capacity of wool to absorb moisture at higher air humidity, and release it to the surroundings with lower air humidity content, has many advantages, because wool acts as a moisture buffer and regulator [5,8].

Although the insulating properties of sheep wool have been analyzed in several studies [8,9,10,11], there is still a need to provide this material with a more stable structure by hot or cold pressing [10] or mixing it with polymers. In this regard, synthetic polymers, such as polyester [12] or epoxy [13] are used in combination with wool fibers, while there are not many studies on natural matrices [14]. To the best of our knowledge, this is the first study regarding an acoustic panel prepared with soy protein and sheep wool fiber.

Sheep wool has been commercialized in different formats for its usage as an acoustic and thermal insulator, combed or as felt, in soft or semirigid panels, or as self-standing panels configured by a thermochemical treatment. In order to commercialize wool in the form of sheets or blankets [15], wool is combined with polymers such as PLA or PET, but these polymers subtract the ecological value of the solution, because they are neither bio-based nor compostable. In this regard, the novelty of this study lies in the combination of two natural raw materials, both of them valorized from residues, to produce biocomposites for acoustic isolation. In this way, manufacturing composites that add value to waste and come entirely from renewable resources have important benefits from the environmental and social perspectives. In this context, the objective of this study was to develop a composite with sheep wool and a bio-based matrix, in particular, soy protein, which is obtained as a by-product from soy oil production. Additionally, glycerol, a by-product of biodiesel production, was used as a plasticizer. Finally, biocomposites were manufactured by freeze drying in order to produce a porous structure that is appropriate for efficiently absorbing sound.

## 2. Materials and Methods

### 2.1. Materials

Raw sheep wool (Latxa breed) was obtained from a local producer (Ametzaga de Zuia, Araba, Spain). This type of wool is predominant in the Basque Country and Navarre in northern Spain. Latxa wool is coarse and, consequently, its use in textile applications has sharply declined over the years. Soy protein isolate (SPI, PROFAM 974), supplied by ADM Protein Specialities Division (Amsterdam, The Netherlands), was used as the polymeric matrix. According to the information provided by the supplier, SPI has a minimum 90% protein content on a dry basis; a maximum of 5% moisture, 4% fat, and 5% ash; and its isoelectric point was 4.6. Finally, glycerol (purity of 99.01%), obtained from Panreac (Barcelona, Spain), was used as a plasticizer.

### 2.2. Sample Preparation

Raw wool was immersed in water at 45 °C for 15 min. Afterward, wool was rinsed and immersed in clean water again at the same temperature for another 15 min. In total, the process was repeated five times to remove dirt and grease. Then, wool was left to dry and stored in a chamber at 25 °C and 50% relative humidity until use.

We mixed 15 g of SPI and the corresponding amount of wool to achieve 7, 10, 15, or 20 wt % wool in the biocomposite (on SPI dry basis), with 500 mL of distilled water. Mixtures were heated at 80 °C at 150 rpm with a magnetic stirrer for 30 min. Then, 20 wt % glycerol (based on SPI dry basis) was added, and the pH was adjusted to 10 (NaOH 1 M). The resulting dispersions were maintained at 80 °C for other 30 min under stirring at 150 rpm. After that, dispersions were poured into molds and frozen at −23 °C for 48 h. Then, samples were freeze dried (Alpha 1–4 LD freeze-dryer Martin Chirst, Thermo Fisher) to obtain wool/soy protein biocomposites, designated as SPI7, SPI10, SPI15, and SPI20 as a function of wool content. All biocomposites were conditioned in a controlled environment chamber at 25 °C and 50% relative humidity before testing.

### 2.3. Sound Absorption Measurements

**Figure 1:**
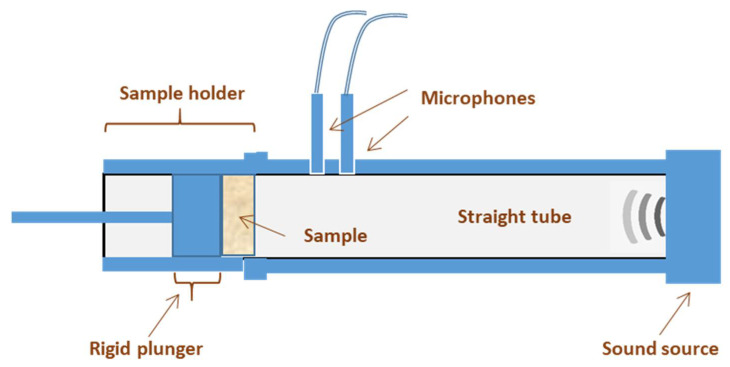
Scheme of the measurement system used to determine the sound absorption coefficient.

A Spectronics ACUPRO Measurement System (NDT Instruments) (Figure 1), with a Kundt’s impedance tube, was used to determine the sound absorption coefficient at normal incidence, using the transfer function method [16] and according to ASTM E-1050. The Kundt’s tube had an inside diameter of 34.9 mm and two 12.7 cm microphones available for measurements in the 50–5700 Hz range. For each composition, 8 tests were carried out to determine the absorption coefficient at normal incidence. The orientation of the samples positioned toward the microphone was alternated for the tests. For each test, the sample was taken out from the holder and put back in it, to avoiding stress and deformation of the sample when placed inside the holder.

### 2.4. Moisture Content (MC)

Samples were weighed (w_0_) and then dried in an oven at 105 °C for 24 h. After this, samples were reweighed (w_1_) to determine their MC according to the following formula:

(1) $$\mathrm{MC}\left(\%\right)=\frac{w_{0}-w_{1}}{w_{0}}\cdot 100$$

### 2.5. Scanning Electron Microscopy (SEM)

SEM analysis was carried out with a Hitachi S-4800 scanning electron microscope. Prior to observation, samples were mounted on a metal stub with double-sided adhesive tape and coated under vacuum with gold, using a JEOL fine-coat ion sputter JFC-1100, in an argon atmosphere. All samples were examined using an accelerating voltage of 10 kV.

### 2.6. X-ray Diffraction (XRD)

XRD analysis was performed with a diffraction unit (PANalytic Xpert PRO, Malvern Instruments, Mavern, Spain) operating at 40 kV and 40 mA. The radiation was generated from a Cu-Kα (λ = 15.18 Ȧ) source. The diffraction data were obtained from 2θ values from 2° to 50°, where θ is the incidence angle of the X-ray beam on the sample.

### 2.7. Fourier Transform Infrared (FTIR) Spectroscopy

FTIR spectra were obtained on a Nicolet Nexus 380 FTIR spectrometer using ATR Golden Gate (Specac, Barcelona, Spain). A total of 32 scans were performed at a resolution of 4 cm^−1^ in the wavenumber range from 800 to 4000 cm^−1^.

### 2.8. Statistical Analysis

Data were subjected to one-way analysis of variance (ANOVA) through SPSS (SPSS Statistic 25.0). Post hoc multiple comparisons were determined by Tukey’s test with the level of significance set at *p* < 0.05.

## 3. Results and Discussion

**Figure 2:**
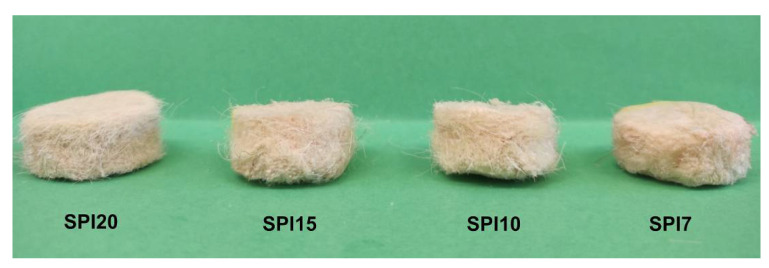
Photo of SPI20, SPI15, SPI 10, and SPI7 biocomposites.

A visual depiction of the Latxa sheep wool/SPI biocomposites developed in this study is presented in Figure 2. As can be observed, all samples displayed good homogeneity and aspect ratio.

**Figure 3:**
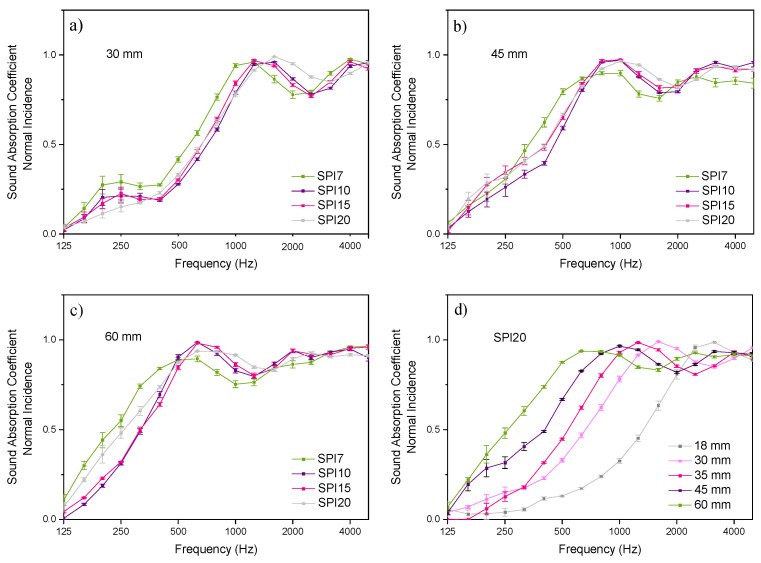
Sound absorption coefficients at normal incidence for SPI7, SPI10, SPI15, and SPI20 samples that were (**a**) 30, (**b**) 45, and (**c**) 60 mm thick. (**d**) Sound absorption coefficient at normal incidence for SPI20 sample as a function of thickness (18, 30, 35, 45, and 60 mm).

In order to explore the acoustic performance of these wool/SPI biocomposites, the sound absorption coefficient was determined at normal incidence at a 100–5000 Hz frequency range for SPI7, SPI10, SPI15, and SPI20 samples as a function of sample thickness. The values are provided in Tables S1 and S2. Representative curves for the samples with 7, 10, 15, and 20 wt % wool are shown in Figure 3a–c as a function of sample thickness (30, 45, and 60 mm).

All the curves show a similar behavior, especially those for wool contents from 10 to 20 wt %. For the samples that were 30 mm thick, three peaks could be observed around 250, 1500, and 4000 Hz. The two latter peaks were the most significant, and they displayed values above 0.9. Regarding the two peaks in Figure 3b,c, these peaks shifted toward lower frequencies when the sample thickness increased, as expected for porous absorbers. In particular, the peaks appeared around 1000 and 3000 Hz for the 45 mm thick samples, while two peaks appeared around 700 and 2000 Hz for the samples that were 60 mm thick. A more thorough analysis as a function of thickness (18, 30, 35, 45, and 60 mm) was performed for the samples with the highest content of wool (SPI20), and the curves are shown in Figure 3d. As previously observed in Figure 2a–c, the maximum of the peak shifted toward lower frequencies as the sample thickness increased from 18 to 60 mm.

The sound absorption coefficients at normal incidence achieved promising values of 0.90–0.99 in all cases. For the sake of comparison, the values measured in this study for sheep wool/SPI biocomposites are similar to those found for sheep wool/PET composites of similar thicknesses [17] and to those observed for glass wool in the case of 50 mm thickness [18]. The values measured for the wool/SPI biocomposites that were 30 mm thick are higher than those found for glass wool with the same thickness, highlighting the potential of the panels developed in this study to provide the same insulating properties with a considerable smaller amount of material. The comparison with the sound absorption coefficients of rock wool and polyurethane foams [19] also indicates the competitiveness of sheep wool/SPI composites.

In order to provide the sound absorption performance at low, middle, and high frequencies at thicknesses of 30, 45, and 60 mm, the arithmetic means of the absorption coefficients at these frequency bands are given in Table 1. In general, these wool/SPI samples were good absorbers, with absorption coefficient values above 0.87 at middle frequencies (1000–2500 Hz bands) and above 0.90 for high frequencies (3150–5000 Hz bands) in all cases, except for SPI7, which showed no significant difference (*p* > 0.05). At low frequencies (100–800 Hz bands), only SPI7 and SPI20 at a 60 mm thickness showed values above 0.5, but no significance difference (*p* > 0.05) was found.

Comparing the composites with sheep wool fibers with those with other natural fibers, the wool/SPI composite showed good acoustic performance at low thickness (around 30 mm) and frequencies around 1000 Hz [3]. Although the results of thermal conductivity coefficient were discrete, the values of the sound absorption coefficient at normal incidence achieved in this study indicated the potential of these fully bio-based composites as alternative materials for acoustic insulating panels. Further analyses to measure the sound absorption coefficient at diffuse incidence with large-area samples (10–12 m^2^) in a reverberating chamber, according to ISO 354:2003, are needed to show the effect of refracted sound waves within the material volume [20,21].

Regarding the effect of moisture content (MC) on the thermal performance of building insulating materials has been the subject of numerous studies in the literature, which have reported an increase in thermal conductivity as moisture content rises due to the fact that water is a worse thermal insulator than air. However, few studies have been conducted on the effect of MC on the acoustic performance [22]. The values of MC measured for the Latxa sheep wool/SPI biocomposites developed in this study are summarized in Table 2. As can be seen, MC decreased (*p* < 0.05) as wool content increased, but the absorption coefficients did not seem to be correlated to the MC, because no clear trend was visible.

In the case of natural fibers, different correlations between average coefficients and moisture content have been found. For kenaf fibers, this correlation was positive and, thus, the increase in moisture content led to an increase in the average absorption coefficient. For cork, the correlation was negative, and average absorption coefficient decreased as moisture content increased [22]. As mentioned above, no clear correlation between the absorption coefficient and MC were found for the sheep wool/SPI composites.

**Figure 4:**
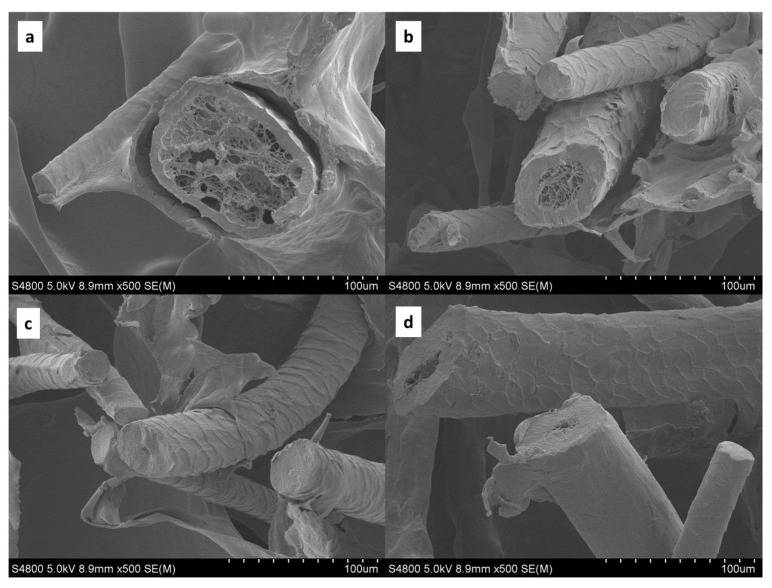
SEM micrographs with magnification of 100 µm for wool/SPI biocomposites: (**a**) SPI7, (**b**) SPI10, (**c**) SPI15, and (**d**) SPI20. Scale bar: 100 µm.

**Figure 5:**
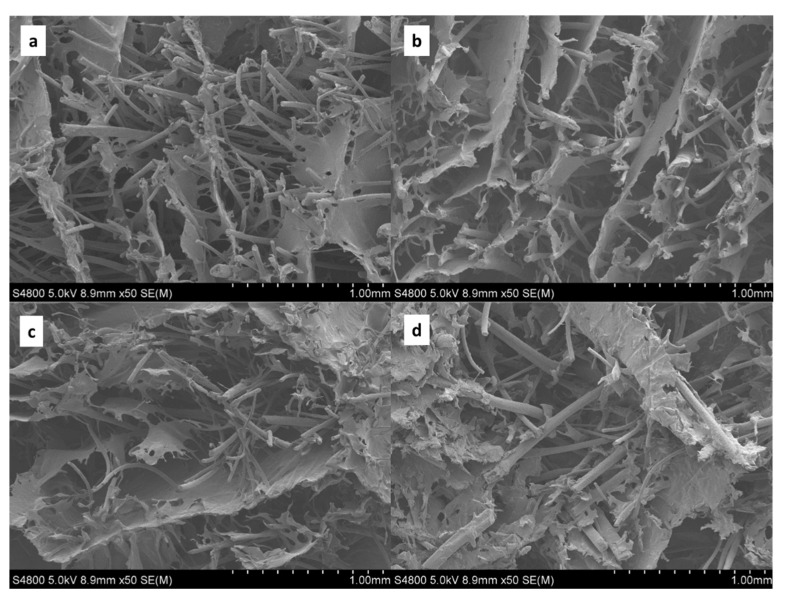
SEM micrographs with magnification of 1 mm for wool/SPI biocomposites: (**a**) SPI7, (**b**) SPI10, (**c**) SPI15, and (**d**) SPI20. Scale bar: 1.00 mm.

In order to relate the acoustic properties of the wool/SPI panels to the microstructure of these biocomposites, scanning electron microscopy (SEM) analysis was carried out. As shown by the SEM images provided in Figure 4, some differences appeared in the wool fiber diameter, with values ranging from ~20 to ~100 μm. Although Latxa wool grad is very coarse [5], the fiber diameters measured in this study are smaller than the values (>100 μm) reported in the literature [5]. As shown in Figure 4, the cross-section of the fibers revealed that the thinnest fibers had a homogeneous structure, whereas the coarsest ones displayed porous nuclei that would correspond to the inner channel, which is called the medula [5]. These porous nuclei are surrounded by a thin homogeneous cortex about 10 μm thick. Additionally, the surface of fibers is covered with scales, as also observed for the fibers from other wool breeds [11]. As shown in SEM images with a higher magnification (Figure 5), the SPI displayed a layer structure that surrounded the fibers.

When sound waves reach an absorbing material, part of the wave energy is dissipated as heat by viscous friction mechanisms inside the volume of the material. In general, a reduction in fiber diameter enhances low-frequency sound absorption by providing a more tortuous path and higher surface area that, in turn, increases the air flow resistivity [23]. For coarse fibers, such as Latxa wool, an effective strategy to improve the sound performance is to combine them with other materials. The incorporation of granular materials diminishes free spaces within and between fibers, improving sound absorption. Although the matrix used in this study was not granular, the freeze-drying process employed provided a similar filling of spaces between fibers, in the form of micropores that could improve performance. A porous structure is closely related to sound absorption performance because the distribution of pathways have a great influence on the sound energy dissipation [24].

**Figure 6:**
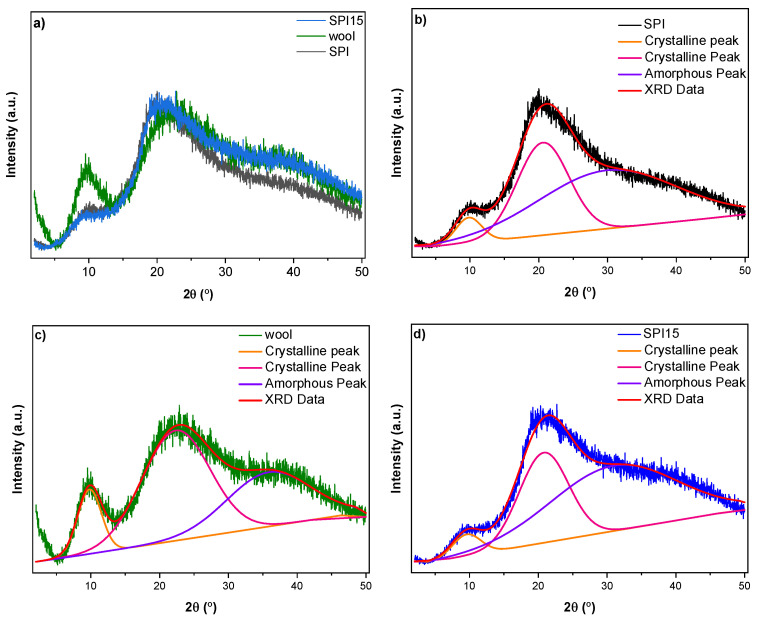
X-ray diffraction patterns of (**a**) SPI, wool, and SPI15, as well as deconvoluted peaks for (**b**) SPI, (**c**) wool, and (**d**) SPI15.

For a further analysis of the structure of the sheep wool/SPI biocomposites, XRD analysis was carried out. For a more clear comparison between samples, the XRD patterns for only Latxa sheep wool, SPI, and SPI15 are shown in Figure 6a. As can be seen, a broad band appeared at 2θ~22° for all the samples, as well as a peak with a lower intensity at 2θ~10°. The peak located at 22° corresponds to β-sheet conformation, while the small peak at 10° is related to α-helix conformation [11]. This peak at 10° showed lower intensity for wool/SPI biocomposites than for neat sheep wool, indicating the more amorphous structure of the biocomposites. To further assess the amorphous/crystalline structure, these two peaks were deconvoluted, and results are shown in Figure 6b–d. As a result of deconvolution, three bands were observed, centered at 2θ~10° and 2θ~22°, related to the crystalline region, and a flat band around 2θ~30°, corresponding to the amorphous phase [11].

The values of the bands resulting from the deconvolution of XRD peaks in Figure 6 are summarized in Table 3. As can be seen, wool/SPI composites showed lower crystallinity than neat sheep wool, probably due to the interactions between the wool and SPI.

**Figure 7:**
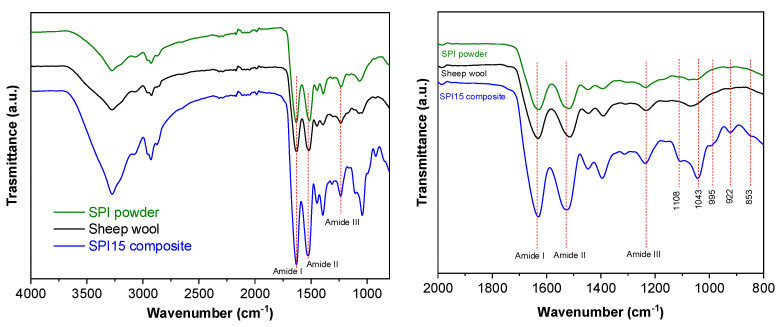
FTIR spectra of neat SPI, neat wool, and SPI15 biocomposite from (**a**) 4000 to 800 cm^−1^ and (**b**) from 2000 to 800 cm^−1^.

In order to assess the interactions between the wool and soy protein, FTIR analysis was carried out. The FTIR spectra for neat sheep wool, neat SPI, and SPI15 biocomposites are plotted in Figure 7. The band at 3500–3000 cm^−1^ corresponds to free and bound O-H and N-H groups (Figure 7a), indicating the ability to form hydrogen bonding with the polar groups in proteins [25,26]. The vibration modes of peptide bonds are represented by amide bands (Figure 7b): amide I around 1630 cm^−1^, due to the C=O stretching vibration; amide II, around 1514 cm^−1^, attributed to the coupling of N-H bending and C-N stretching; and amide III, around 1232 cm^−1^, related to C-N stretching coupled with plane N-H bending. Because wool mainly contains keratin [27], all samples showed the abovementioned amide bands. Additionally, the bands corresponding to glycerol were observed in the range of 853–1108 cm^−1^. Bands at 853, 922 and 995 cm^−1^ correspond to the vibration of C-C skeletons, the band at 1043 cm^−1^ is associated with C-O bond stretching, and the band at 1108 cm^−1^ is attributed to the stretching of C-O in the central carbon [16]. The most relevant change among the three FTIR spectra was related to the relative intensity between the amide I and II bands. This relative intensity was similar for neat materials, while the amide I band intensity was higher than that of amide II for the biocomposites. This change was indicative of physical interactions among wool and soy protein, probably through hydrogen bonds among the polar groups of both proteins.

## 4. Conclusions

This study revealed that the process of freeze drying provided sheep wool/SPI biocomposites with an appropriate microstructure for a good acoustic absorption performance at a wide range of frequencies from 100 to 5000 Hz. The measured sound absorption coefficients were above 0.9 for a wider range of frequencies as the thickness increased from 18 to 60 mm, regardless the wool content in the biocomposite. This behavior was related to the fibrillary microstructure of the biocomposite, which showed fiber diameters lower than 100 µm, resulting in a homogeneous structure. Additionally, the incorporation of wool led to a more amorphous structure, as shown by XRD analysis, probably due to the physical interactions of hydrogen bonding between soy protein and wool. The values of the sound absorption coefficient found in this study are similar to those of conventional acoustic absorbers available on the market, such as glass wool and polyurethane foams, and even better under some specific conditions. This approach highlights the relevance of these novel biocomposites based on a biopolymeric matrix reinforced with natural fibers to valorize residues with the aim of developing more sustainable materials.

## Figures and Tables

**Table 1:** Average and standard deviation of sound absorption coefficients at normal incidence (α) for SPI7, SPI10, SPI15, and SPI20 biocomposites at low, middle, and high frequency bands.

Average α	30 mm Thick				45 mm Thick				60 mm Thick			
	SPI7	SPI10	SPI15	SPI20	SPI7	SPI10	SPI15	SPI20	SPI7	SPI10	SPI15	SPI20
Low frequencies (100–800 Hz bands)	0.30 ^a^	0.22 ^a^	0.24 ^a^	0.26 ^a^	0.44 ^a^	0.37 ^a^	0.41 ^a^	0.42 ^a^	0.56 ^a^	0.46 ^a^	0.46 ^a^	0.52 ^a^
Middle frequencies (1000–2500 Hz bands)	0.87 ^b^	0.87 ^b^	0.87 ^b^	0.90 ^b^	0.83 ^b^	0.87 ^b^	0.88 ^b^	0.89 ^b^	0.82 ^b^	0.87 ^b^	0.87 ^b^	0.88 ^b^
High frequencies (3150–5000 Hz bands)	0.94 ^c^	0.90 ^c^	0.91 ^c^	0.90 ^c^	0.85 ^c^	0.95 ^c^	0.92 ^c^	0.93 ^c^	0.95 ^c^	0.93 ^c^	0.94 ^c^	0.91 ^c^

Two means followed by the same letter in the same column are not significantly (*p* > 0.05) different according to Tukey’s multiple range test.

**Table 2:** Moisture content (MC) values of SPI7, SPI10, SPI15, and SPI20 biocomposites.

Sample	MC (%)
SPI7	18.8 ± 0.4 ^a^
SPI10	17.0 ± 0.4 ^b^
SPI15	16.0 ± 0.2 ^b^
SPI20	14.3 ± 0.5 ^c^

Two means followed by the same letter in the same column are not significantly (*p* > 0.05) different according to Tukey’s multiple range test.

**Table 3:** Values of the area under the deconvoluted XRD peaks for neat sheep wool, neat SPI, and SPI15 biocomposites.

Area (%)	2θ~10°	2θ~22°	2θ~30°
Wool	11.3	55.3	33.4
SPI	4.9	36.2	58.9
SPI15	4.6	33.9	61.5

## Data Availability

Data is contained within the article and supplementary material.

## Supplementary Materials

The following supporting information can be downloaded at: https://www.mdpi.com/article/10.3390/polym14235231/s1, Table S1: Sound absorption coefficients at normal incidence of SPI7, SPI10, and SPI15 biocomposites as a function of thickness (30, 45, and 60 mm); Table S2: Sound absorption coefficients at normal incidence of wool/SPI composite SPI20 biocomposite as a function of sample thickness (18, 30, 35, 45, and 60 mm).
