# Supplementary material for: Sustainable Sheep Wool/Soy Protein Biocomposites for Sound Absorption
Source: Polymers (Basel). 2022 Dec 1;14(23):5231. doi: 10.3390/polym14235231 (PMC9740274; doi:10.3390/polym14235231)
Supplement: Supplementary file 1 [file polymers-14-05231-s001.zip › polymers-2060537-supplementary.pdf]

Table S1. Sound absorption coefficients at normal incidence of SPI7, SPI10, and SPI15 biocomposites as a function of thickness (30, 45, and 60 mm).

	SPI7			SPI10			SPI15		
Thickness (mm)	30	45	60	30	45	60	30	45	60
f (Hz)									
100	0.018±0.004	0.038±0.009	0.06±0.02	0.03±0.02	0.02±0.02	0±0	0.05±0.03	0.012±0.07	0±0
125	0.03±0.02	0.06±0.01	0.11±0.03	0.022±0.008	0.032±0.005	0.007±0.006	0.03±0.01	0.02±0.01	0.044±0.007
160	0.14±0.03	0.16±0.03	0.30±0.02	0.08±0.02	0.12±0.03	0.084±0.007	0.09±0.03	0.15±0.04	0.122±0.003
200	0.27±0.05	0.23±0.05	0.44±0.04	0.20±0.04	0.19±0.04	0.188±0.009	0.17±0.03	0.27±0.04	0.231±0.005
250	0.29±0.04	0.31±0.03	0.55±0.03	0.21±0.04	0.26±0.05	0.31±0.01	0.23±0.04	0.35±0.04	0.321±0.003
315	0.27±0.02	0.47±0.03	0.74±0.01	0.21±0.02	0.33±0.02	0.49±0.02	0.19±0.01	0.41±0.02	0.50±0.01
400	0.274±0.006	0.62±0.03	0.840±0.006	0.188±0.006	0.39±0.01	0.70±0.02	0.197±0.008	0.48±0.02	0.64±0.01
500	0.42±0.02	0.80±0.02	0.89±0.01	0.278±0.008	0.59±0.01	0.90±0.02	0.302±0.006	0.65±0.01	0.85±0.01
630	0.56±0.01	0.868±0.009	0.89±0.02	0.417±0.009	0.803±0.007	0.987±0.002	0.46±0.01	0.839±0.007	0.982±0.004
800	0.76±0.02	0.898±0.009	0.82±0.02	0.583±0.009	0.959±0.004	0.92±0.01	0.64±0.01	0.967±0.008	0.959±0.006
1000	0.94±0.01	0.90±0.01	0.75±0.02	0.790±0.005	0.970±0.001	0.83±0.01	0.84±0.01	0.973±0.003	0.86±0.01
1250	0.961±0.008	0.78±0.02	0.76±0.02	0.946±0.004	0.877±0.003	0.80±0.01	0.969±0.005	0.89±0.01	0.80±0.01
1600	0.86±0.02	0.76±0.02	0.84±0.02	0.960±0.007	0.789±0.003	0.87±0.01	0.939±0.005	0.82±0.01	0.8±0.02
2000	0.78±0.02	0.85±0.01	0.86±0.02	0.866±0.007	0.795±0.006	0.940±0.009	0.832±0.008	0.82±0.01	0.94±0.01
2500	0.79±0.01	0.88±0.02	0.88±0.01	0.781±0.005	0.914±0.007	0.90±0.01	0.770±0.008	0.91±0.01	0.92±0.01
3150	0.90±0.01	0.85±0.02	0.931±0.003	0.815±0.005	0.958±0.005	0.931±0.008	0.849±0.009	0.936±0.006	0.92±0.01
4000	0.976±0.003	0.86±0.02	0.959±0.006	0.938±0.008	0.929±0.008	0.949±0.009	0.963±0.005	0.92±0.01	0.95±0.01
5000	0.949±0.005	0.84±0.03	0.964±0.007	0.96±0.01	0.958±0.004	0.90±0.02	0.923±0.007	0.92±0.01	0.96±0.01

Table S2. Sound absorption coefficients at normal incidence of wool/SPI composite SPI20 biocomposite as a function of sample thickness (18, 30, 35, 45, and 60 mm).

f (Hz)	Thickness (mm)	18	30	35	45	60
100		0.03±0.02	0.0049±0.0008	0.03±0.01	0.03±0.01	0.03±0.01
125		0.06±0.04	0±0	0.04±0.01	0.04±0.01	0.07±0.02
160		0.03±0.02	0.0004±0.0003	0.07±0.01	0.20±0.04	0.22±0.01
200		0.03±0.03	0.06±0.03	0.11±0.02	0.29±0.05	0.36±0.05
250		0.04±0.02	0.13±0.03	0.15±0.03	0.32±0.03	0.48±0.03
315		0.055±0.009	0.181±0.007	0.175±0.007	0.41±0.02	0.60±0.02
400		0.12±0.01	0.316±0.005	0.229±0.008	0.490±0.006	0.738±0.008
500		0.131±0.005	0.447±0.006	0.33±0.01	0.669±0.006	0.875±0.005
630		0.173±0.008	0.622±0.009	0.47±0.01	0.826±0.004	0.937±0.002
800		0.240±0.008	0.80±0.001	0.62±0.02	0.924±0.003	0.935±0.004
1000		0.33±0.01	0.929±0.006	0.78±0.02	0.965±0.005	0.914±0.004
1250		0.45±0.02	0.985±0.001	0.915±0.005	0.945±0.004	0.848±0.006
1600		0.63±0.03	0.944±0.006	0.990±0.001	0.864±0.003	0.832±0.009
2000		0.81±0.03	0.853±0.006	0.951±0.006	0.819±0.005	0.895±0.008
2500		0.96±0.01	0.808±0.004	0.877±0.006	0.863±0.006	0.927±0.005
3150		0.986±0.002	0.856±0.005	0.848±0.005	0.935±0.004	0.905±0.006
4000		0.93±0.01	0.931±0.009	0.897±0.006	0.928±0.008	0.917±0.008
5000		0.887±0.007	0.91±0.02	0.955±0.005	0.92±0.01	0.911±0.007
